# Ointments and Oleates Especially in Diseases of the Skin

**Published:** 1890-12

**Authors:** 


### BOOK NOTICES.

OINTMENTS AND OLEATES ESPECIALLY IN DISEASES OF THE SKIN. By John V. Shoemaker, A. M., M. D., Professor of Materia Medica, Pharmacology, Therapeutics, and Clinical Medicine, etc., Medico-Chirurgical College of Philadelphia. Philadelphia and London: F. A. Davis, 1890.

This work is the revised and enlarged second edition, which shows there is a demand for it. Viewed from the standpoint of allopathy we should consider it a valuable work as it gives the officinal ointments of the pharmacopœia of Austria, Britain, France, Germany, Italy, Spain, Mexico, and Chili. And then the more recent oleates receive full notice, and altogether grease is well represented.

The author concisely concludes his preface as follows: "The reader may thus obtain a conspectus of the whole subject of inunction as it exists to-day in the civillized world. In all cases the mode of preparation is given, and the therapeutical applications described *seriatim*, in so far as may be done without needless repetition."

We, of the homœopathic school, can make no use of the various mixtures described, as we have something better, but we may commend the book for the accuracy and clearness of its recipes and for its neat binding and the excellence of the printers' work.

